# CU06-1004 enhances vascular integrity and improves cardiac remodeling by suppressing edema and inflammation in myocardial ischemia–reperfusion injury

**DOI:** 10.1038/s12276-021-00720-w

**Published:** 2022-01-07

**Authors:** Haiying Zhang, Hyeok Kim, Bong Woo Park, Minyoung Noh, Yeomyeong Kim, Jeongeun Park, Jae-Hyun Park, Jin-Ju Kim, Woo-Sup Sim, Kiwon Ban, Hun-Jun Park, Young-Guen Kwon

**Affiliations:** 1 Department of Biochemistry, College of Life Science and Biotechnology, Yonsei University, Seoul, 120-749 Republic of Korea; 2 R&D Department, Curacle Co. Ltd, Seongnam-si, Republic of Korea; 3 Department of Medical Life Science, College of Medicine, The Catholic University of Korea, Seoul, 06591 Republic of Korea; 4 Department of Biomedical Sciences, City University of Hong Kong, Kowloon Tong, 999077 Hong Kong; 5 Division of Cardiology, Department of Internal Medicine, The Catholic University of Korea, Banpo-daero 222, Seocho-gu, Seoul, 137701 Republic of Korea

**Keywords:** Drug development, Heart failure

## Abstract

Ischemia–reperfusion (I/R) injury accelerates the cardiomyocytes (CMs) death by oxidative stress, and thereby deteriorates cardiac function. There has been a paradigm shift in the therapeutic perspective more towards the prevention or amelioration of damage caused by reperfusion. Cardiac microvascular endothelial cells (CMECs) are more vulnerable to reperfusion injury and play the crucial roles more than CMs in the pathological process of early I/R injury. In this study, we investigate that CU06-1004, as a vascular leakage blocker, can improve cardiac function by inhibiting CMEC’s hyperpermeability and subsequently reducing the neutrophil’s plugging and infiltration in infarcted hearts. CU06-1004 was delivered intravenously 5 min before reperfusion and the rats were randomly divided into three groups: (1) vehicle, (2) low-CU06-1004 (1 mg/kg, twice at 24 h intervals), and (3) high-CU06-1004 (5 mg/kg, once before reperfusion). CU06-1004 treatment reduced necrotic size and cardiac edema by enhancing vascular integrity, as demonstrated by the presence of intact junction proteins on CMECs and surrounding pericytes in early I/R injury. It also decreased the expression of vascular cell adhesion molecule 1 (VCAM-1) on CMECs, resulting in reduced infiltration of neutrophils and macrophages. Echocardiography showed that the CU06-1004 treatment significantly improved cardiac function compared with the vehicle group. Interestingly, single high-dose treatment with CU06-1004 provided a greater functional improvement than repetitive low-dose treatment until 8 weeks post I/R. These findings demonstrate that CU06-1004 enhances vascular integrity and improves cardiac function by preventing lethal myocardial I/R injury. It can provide a promising therapeutic option, as potential adjunctive therapy to current reperfusion strategies.

## Heart attack: Drug boosts survival of heart cells during treatment

A drug that can block excess inflammation and leakage from blood vessels shows promise in boosting the survival of heart cells following treatment for heart attack. Reperfusion therapy aims to restore blood flow around or through blocked arteries after a heart attack. However, in some cases the treatment can trigger the overproduction of reactive oxygen species, damaging the endothelium (lining of blood vessels) and further injuring heart muscle cells. Hun-Jun Park (Catholic University) and Young-Guen Kwon (Yonsei University), both in Seoul, South Korea, and co-workers, have demonstrated that the drug CU06-1004, used successfully in animal trials for other conditions, can enhance cardiac integrity and function following reperfusion in rats. A single high dose of CU06-1004 inhibited leakage from heart microvascular endothelial cells, reducing the build-up of fluid in tissues and limiting tissue death.

## Introduction

Acute myocardial infarction (AMI) is the leading cause of death and critical disability worldwide^1^. The standard therapy, which consists of timely myocardial reperfusion using primary percutaneous coronary intervention (PCI), has been considered the most effective therapeutic strategy in patients with AMI^2^. Reperfusion of the occluded culprit arteries within a short time can reduce ischemic injury and prevent necrosis of cardiomyocytes (CMs). However, several pieces of evidence have shown that reperfusion of the ischemic myocardium causes further damage, accounting for up to 50% of the final infarct size. Since reperfusion itself has reduced MI‑related mortality, therapeutic perspectives have undergone a paradigm shift toward the prevention or amelioration of damage caused by reperfusion^3^. Until now, despite a significant amount of research as well as encouraging preclinical results with multiple agents, most of the clinical trials to prevent ischemia–reperfusion (I/R) injury have been disappointing^4–9^; thus, it is important to identify and develop therapies that potentially reduce infarct size.

Cardiac microvascular endothelial cells (CMECs) are more vulnerable than CMs to reperfusion injury and play more crucial roles in the pathological process of early I/R injury^10^. Reactive oxygen species (ROS), such as superoxide and its metabolites, affect vascular homeostasis and function via multiple signaling pathways. The production of ROS peaks during the first 2 min to 10 min after myocardial reperfusion. Excessive ROS cause hyperpermeability by destabilizing the endothelial barrier, composed of proteins that normally maintain tight junctions between CMECs. Simultaneously, adhesion molecules such as intercellular adhesion molecule 1 (ICAM-1), vascular cell adhesion molecule 1 (VCAM-1), and E-selectin are upregulated on CMECs, which causes neutrophils to plug capillaries and infiltrate the infarcted tissue. Neutrophil infiltration of the injured tissue leads to the production of more ROS than CMECs, which significantly contributes to severe myocardial damage via inflammatory responses^3^.

In our previous study, we demonstrated that CU06-1004 (also known as Sac-1004) enhanced endothelial cell survival under serum-deprivation conditions and prevented endothelial barrier disruption induced by vascular endothelial growth factor (VEGF), histamine, thrombin, and IL-1β in vitro^11,12^. It also inhibited ICAM-1 and VCAM-1 expression by inhibiting nuclear factor-κB (NF-κB) activation^11^. CU06-1004 exerts therapeutic effects by inhibiting vascular leakage and inflammation in various animal models, such as diabetic retinopathy, stroke, cancer, and inflammatory bowel disease^11–13^. In the heart, CMECs and CMs are in close proximity and interact through the secretion of paracrine signals, as well as through direct cell-to-cell contact in normal and pathological conditions^14^. In the early stages of reperfusion, damaged CMECs release soluble proapoptotic factors, which induce the apoptosis of CMs^15^. On the other hand, activated CMECs upregulate endothelial nitric oxide synthase (eNOS) expression, which is involved in the survival and contractility of CMs^16,17^. Given the correlation between CMECs and CMs in the pathophysiology of I/R injury, we hypothesize that intravenous injection of CU06-1004 at the time of reperfusion can enhance the survival of CMs by protecting the vascular integrity of damaged CMECs and thereby improve cardiac function and remodeling in I/R-injured hearts.

## Materials and methods

### Drugs

CU06-1004 was synthesized as described previously^11,12^. Briefly, CU06-1004 was synthesized via tetrahydropyran deprotection and subsequent glycosidation with 4,6-di-O-acetyl-2,3-didieoxyhex-2-enopyran in the presence of acid.

### Cell culture

Human CMECs (Cat. #6000) were purchased from ScienCell Research Laboratories. Cells were grown in 2% gelatin-coated dishes and maintained in endothelial cell medium (ECM, Cat. #1001) at 37 °C under a humidified 95–5% (v/v) mixture of air and CO_2_. All experiments were carried out with confluent cells between the third and sixth passages. Human CMs (Cat. #C-12810) were purchased from PromoCell and cultured in Ready-to-Use Myocyte Growth Medium (PromoCell). Cultures were maintained at 37 °C and 5% CO_2_. All experiments were conducted in passages 3–6. Coculture was performed using a permeable 24-well Transwell plate (Corning) consisting of an upper insert and a lower chamber to determine the effect of ECs on CM injury. Human CMECs were seeded at a density of 4.5 × 10^4^ cells/well in the upper insert. Human CMs were seeded at a density of 1.5 × 10^4^ cells/well in the lower chamber. The cells were left undisturbed for 24 h so that they could attach.

### Hypoxia and reoxygenation in vitro

Human CMECs were treated with different concentrations of CU06-1004 or vehicle (DMSO) and cultivated in DMEM (low glucose without fetal calf serum), and the cells were then transferred into a sealed Anaero container with an Anaero pack for 16 h to initiate hypoxia. Subsequently, the medium was replaced with endothelial cell medium, and the cells were cultivated for an additional 24 h under normoxic conditions before being subjected to various analyses.

### Quantitative real-time reverse transcription-polymerase chain reaction

cDNA was synthesized using Moloney murine leukemia virus reverse transcriptase. Quantitative real-time polymerase chain reaction (qRT–PCR) was performed with SYBR Green in a Piko real-time PCR detection system (Thermo Scientific). The primers used were as follows:

ICAM-1, 5′-CCCAAGTTGTTGGGCATAGA-3′ and 5′-CCCATCAGGGCAGTTTGAAT-3′;

VCAM-1, 5′-CATCCACAAAGCTGCAAGAAG -3′ and 5′- AGGTGCTGTAGATTCCCATTATC-3′;

E-SELE, 5′-GTGTATGTCCTCTGGAGAATGG-3′ and 5′ GAACCCATTGGCTGGATTTG-3′;

MCP-1, 5′-TCATAGCAGCCACCTTCATTC-3′ and 5′ CTCTGCACTGAGATCTTCCTATTG-3′;

TNF-α, 5′-CATCTACTCCCAGGTCCTCTT-3′ and 5′ TTGACCTTGGTCTGGTAGGA-3′;

IL-1β, 5′-ATGGACAAGCTGAGGAAGATG-3′ and 5′ CCCATGTGTCGAAGAAGATAGG-3′;

GAPDH, 5′-GGTGTGAACCATGAGAAGTATGA-3′ and 5′-GAGTCCTTCCACGATACCAAAG-3′.

The thermal cycling parameters were as follows: 10 min at 94 °C, 30 amplification cycles (denaturation at 95 °C for 15 s, annealing at 60 °C for 30 s and extension at 72 °C for 30 s) and a final extension step at 72 °C for 30 s.

### MTT assay

Human CMECs were seeded at a density of 3 × 10^4^ cells/well in gelatin-coated 24-well plates and incubated overnight. After the necessary treatment, cells were reacted with 0.1 mg/mL MTT solution and incubated at 37 °C for 4 h. The residual MTT was carefully removed, and the crystals were dissolved in DMSO:ethanol (1:1). The absorbance was measured at 560 nm with a FLUOstar Omega microplate reader.

### Enzyme-linked immunosorbent assay (ELISA)

BNP and TNF-α concentrations in the medium of cocultured human CMECs and CMs were measured by ELISA kits (Elabscience) according to the manufacturer’s instructions. After the reactions, 96-well microplates were read by measuring the OD with an Omega reader at 450 nm.

### ROS measurement

The intracellular ROS levels were measured using the fluorescent dye 2′,7′-dichlorofluorescin diacetate. Human CMECs were treated with CU06-1004 under hypoxic conditions, washed twice, stained with 10 μM DCF-DA for 30 min at 37 °C, and washed twice again. The fluorescence intensity was quantified using a FLUOstar Omega microplate reader.

### FITC-dextran permeability assay

Human CMECs were grown until confluent on the luminal side of filters (0.4-μm pore size; Corning) coated with gelatin in 12-well plates. Cells were cultured and subjected to hypoxic conditions and CU06-1004 treatment as described previously. FITC-dextran (1 mg/mL; Sigma) was added to the upper compartment. The absorbance of the solution in the lower chamber was measured at 492 nm (excitation) and 520 nm (emission) in a FLUOstar Omega microplate reader.

### Myocardial infarction and drug delivery

All animal studies were approved by the Institutional Animal Care and Use Committee (IACUC) of The Catholic University of Korea (approval number: CUMC-2020-0051-01). All animal procedures conformed to the NIH guidelines or the guidelines issued by Directive 2010/63/EU of the European Parliament for the protection of animals used in scientific research. Fisher 344 rats (160–180 g, 8-week-old males, Koatec, Korea) were anesthetized with 2% inhaled isoflurane and intubated with an 18 G intravenous catheter in the trachea. The rats were ventilated with a rodent respirator (Harvard Apparatus). A 37 °C, heating pad was used to maintain their body temperature throughout the operation. The chest was shaved and sterilized with 70% alcohol. Ischemia–reperfusion injury was induced by occluding the LAD artery with a 7-0 Prolene suture for 30 min. The drug was diluted with PBS (500 µL) and intravenously delivered just before reperfusion. The rats were randomly divided into three groups: (1) the vehicle group (treated only with PBS), (2) the low-CU06-1004 group (treated twice with 1 mg/kg at 24 h intervals), and (3) the high-CU06-1004 group (treated once with 5 mg/kg before reperfusion). Then, the chest was closed aseptically, cleansed with 0.9% normal saline solution, and treated with topical antibiotics. After 24 h, the low-CU06-1004 (1 mg/kg) group received the same dose of drug intravenously once again.

### Measurement of myocardial infarct size

Combined Evans blue and triphenyltetrazolium chloride (TTC) staining was performed to determine the early cardioprotective effects of CU06-1004. Rats were anesthetized and ventilated as described previously. After 30 min of ischemia and 48 h of reperfusion, the suture thread around the LAD artery was retied, and Evans blue dye (9% in PBS) was injected intravenously into the rats. After 15 min, the heart was quickly excised and incubated for 10 min at −4 °C. The heart was cut into three slices (each one ~2 mm thick) and incubated with 2% TTC for 30 min at 37 °C in the dark. After being washed three times, the tissue was fixed in 4% paraformaldehyde. The non-infarcted myocardium was stained deep blue with Evans Blue. The viable myocardium was stained red with TTC. The necrotic myocardium appeared white after TTC staining. The area at risk (AAR) and the necrotic area were determined digitally by ImageJ.

### Measurement of cardiac edema

Evans blue staining was performed to determine vascular permeability after CU06-1004 treatment. Rats were anesthetized and ventilated as described previously. After 30 min of ischemia and 48 h of reperfusion, Evans blue dye (2% in PBS) was injected intravenously into the rats and circulated for 4 h. Rats were euthanized, and all blood was immediately washed out with citrate buffer. The hearts were then excised, and Evans blue dye was eluted in formamide for 18 h at 65 °C. The absorbance of Evans blue dye at 620 nm was measured using a Molecular Devices Spectramax 250 Microplate Reader (Marshall Scientific, Hampton, NH, USA).

### Immunofluorescence staining

The heart sections were incubated in blocking solution for 1 h at room temperature and then incubated at 4 °C overnight with one of the following antibodies: rabbit anti-VE-cadherin (1:200; Invitrogen Cat. #36-1900), goat anti-CD31 (1:200; R&D Cat. #AF3628), rabbit anti-zonula occludens-1 (1:200; Invitrogen Cat. #61-7300), rabbit anti-occludin (1:200; Invitrogen Cat. #71-1500), mouse anti-NG2 (1:100; Millipore Cat. #AB5320), rabbit anti-VCAM-1 (1:200; Santa Cruz Cat. #sc-13160), rabbit anti-MPO (1:50; Abcam Cat. #ab9535), mouse anti-CD68 (1:100; Abcam Cat. #ab955), rabbit anti-iNOS (1:200; Abcam Cat. #ab15323), rabbit anti-CD206 (1:100; Abcam Cat. #ab64693), or mouse anti-cTnT (1:200; Abcam Cat. #ab8295). Confocal images were captured at room temperature with ZEN software on an upright confocal microscope (LSM 700; Carl Zeiss) using the predefined ZEN software configurations for Alexa Fluor 546, Alexa Fluor 488, and DAPI.

### H&E staining

At 48 h after I/R injury, the rats were euthanized, and the hearts were harvested. The hearts were fixed in 4% paraformaldehyde overnight, embedded in paraffin, and sectioned into 4-μm sections using a microtome (Leica, RM2255, Germany). The sections were deparaffinized with xylene and rehydrated. The sections were immersed in hematoxylin solution for 10 s and eosin solution for 30 s. Washes were performed between each step. After being washed with tap water, the tissues were dehydrated and mounted. Imaging of sections was performed with Panoramic MIDI.

### TUNEL assay

A terminal deoxynucleotidyl transferase dUTP nick-end labeling kit (Roche; Cat. #11684795910) assay was used to identify apoptosis. The sections were deparaffinized with xylene and rehydrated. The sections were permeabilized with 200 μL of TBS-T for 2 min on ice. The sections were then incubated for 1 h at 37 °C in the dark with staining solution containing deoxynucleotidyl transferase. After being washed washing with PBS, the sections were mounted with DAPI mounting solution (Vector; H-1500).

### Echocardiography

The animals were anesthetized with 2% isoflurane and placed on a heating pad to maintain their body temperature at 37 °C. Serial echocardiography was performed 1, 2, 4, and 8 weeks after treatment using a transthoracic echocardiography system equipped with a 15 MHz L15-7io linear transducer (Affiniti 50 G, Philips) to determine the ejection fraction (EF), fractional shortening (FS), left ventricular internal diameter at end diastole (LVIDd), left ventricular internal diameter at end-systole (LVIDs), septal wall thickness (SWT), and posterior wall thickness (PWT). The operator of the echocardiography system was blinded to the group allocations of the animals during the experiment.

EF (%) = [(LVEDV-LVESV)/LVEDV] × 100

FS (%) = [(LVEDD-LVESD)/LVEDD] × 100

### Hemodynamic measurements

Hemodynamic measurements were performed at the 8-week endpoint, prior to euthanasia. Rats were anesthetized and ventilated as described previously. After thoracotomy without bleeding, the LV apex of the heart was punctured with a 26 G needle, and a 2 F conductance catheter (SPR-838, Millar) was inserted into the LV. LV pressure–volume parameters were continually recorded using a PV conductance system (MPVS Ultra, emka TECHNOLOGIES, Paris, France) coupled to a digital converter (PowerLab 16/35, ADInstruments, Colorado Springs, CO). Load-independent parameters of cardiac function, including the slopes of the end-systolic pressure–volume relationship (ESPVR) and end-diastolic pressure–volume relationship (EDPVR), were measured at different preloads, which were elicited by transient occlusion of the inferior vena cava with a needle holder. Fifty microliters of hypertonic saline (20% NaCl) was injected into the left jugular vein to calculate the parallel conductance after hemodynamic measurements. Blood was collected from the left ventricle into a heparinized syringe and transferred into cuvettes to convert the conductance signal to volume using the catheter. The absolute volume of the rat was defined by calibrating the parallel conductance and the cuvette conductance.

### Masson’s trichrome staining

Eight weeks after treatment, the rats were euthanized, and the hearts were harvested. The hearts were fixed in 4% paraformaldehyde overnight, embedded in paraffin, and sectioned into 4-μm sections starting at the top of the apex using a microtome (Leica, RM2255, Germany). The sections were deparaffinized with xylene and fixed in Bouin’s solution at 56 °C for 90 min. The sections were stained with Weigert’s iron hematoxylin solution for 15 min at room temperature and washed with tap water for 15 min. The sections were then stained with Biebrich scarlet-acid fuchsin solution for 15 min at room temperature. The sections were stained with phosphomolybdic acid for 15 min and then aniline blue for 15 min, followed by incubation in 1% acetic acid for 2 min at room temperature. Washes were performed between each step. Finally, after the sections were mounted, they were imaged with a Pannoramic MIDI. The percentage of fibrotic area relative to the entire left ventricular wall area was quantified using ImageJ software with basic add-ons.

### Capillary density

The sections were deparaffinized with xylene and washed with PBS. The sections were treated with goat anti-CD31 (1:200; R&D Cat. #AF3628) as the primary antibody and incubated at 4 °C overnight. The sections were washed with PBS, stained with donkey anti-goat IgG (1:500; Invitrogen Cat. #A11055) as the secondary antibody, and mounted with DAPI mounting solution (Vector; H-1500). The capillaries were counted in five random fields of view using a fluorescence microscope (Nikon) and expressed as the number of capillaries per square millimeter of tissue area.

### Data analysis

All quantitative data are shown as the means ± SEM unless otherwise indicated. Differences between the two groups were tested for statistical significance using a two-tailed Student’s *t* test. Differences among three or more groups were tested for significance by ANOVA with Bonferroni’s post hoc analysis. The results were considered significant when the *P* value was less than 0.05.

## Results

### CU06-1004 protects against I/R-related myocardial damage by reducing vascular permeability

**Fig. 1:**
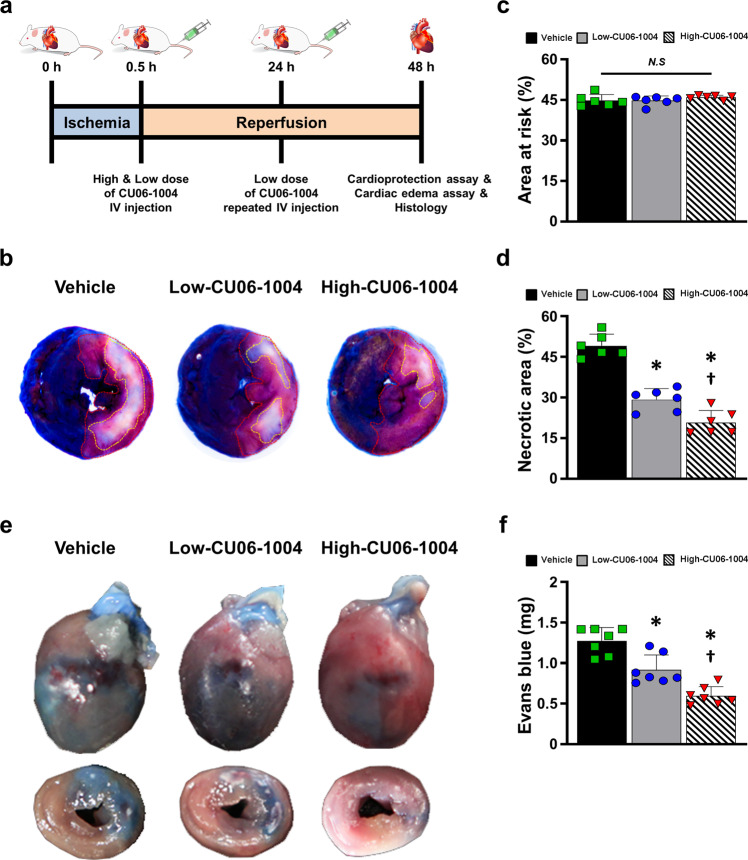
CU06-1004 exerts a cardioprotective effect by reducing vascular permeability in I/R injury. **a** Scheme of the experiment to determine the cardioprotective effect of CU06-1004 in I/R injury. **b** Representative images from the Evans blue staining and TTC assay at 48 h after reperfusion; red dotted line: area at risk; yellow line: necrotic area. **c**, **d** Quantification results of (**c**) area at risk and (**d**) necrotic area. *n* = 6. *P < 0.05 vs. vehicle. ^†^*P* < 0.05 vs. low CU06-1004. N.S. not significant. **e** Representative images of the cardiac edema assay at 48 h after reperfusion. **f** Quantification of Evans blue leakage into interstitial tissue; blue area: extravasation of Evans blue. *n* = 7. *P < 0.05 vs. vehicle. ^†^*P* < 0.05 vs. low CU06-1004. Data are shown as the mean ± SEM.

We investigated the cardioprotective effects of CU06-1004 on infarct size in early I/R injury (Fig. 1a). Combined Evans blue and TTC staining was performed 48 h after reperfusion and showed that CU06-1004 treatment significantly reduced the necrotic area in a dose-dependent manner compared with the vehicle, even though the area at risk (AAR), which indicated the ischemic region under LAD artery ligation, was the same in all groups (Fig. 1b–d). These results suggest that CU06-1004 has cardioprotective effects in preventing lethal myocardial I/R injury, and treatment with a single high-dose administration of CU06-1004 during early myocardial I/R injury was more effective than repeated low-dose administration of this agent.

Subsequently, we determined whether CU06-1004 prevented vascular leakage in the same experimental model using Evans blue dye (2% in PBS). Cardiac edema, which was quantified by extracting Evans blue that had leaked into interstitial tissue, was significantly reduced in the low-CU06-1004 group compared with the vehicle group and further reduced in the high-dose group (Fig. 1e, f). These data suggest that the intravenous administration of CU06-1004 prevents myocardial damage by reducing vascular permeability after early myocardial I/R injury.

### CU06-1004 prevents neutrophil influx by strengthening EC junctions

**Fig. 2:**
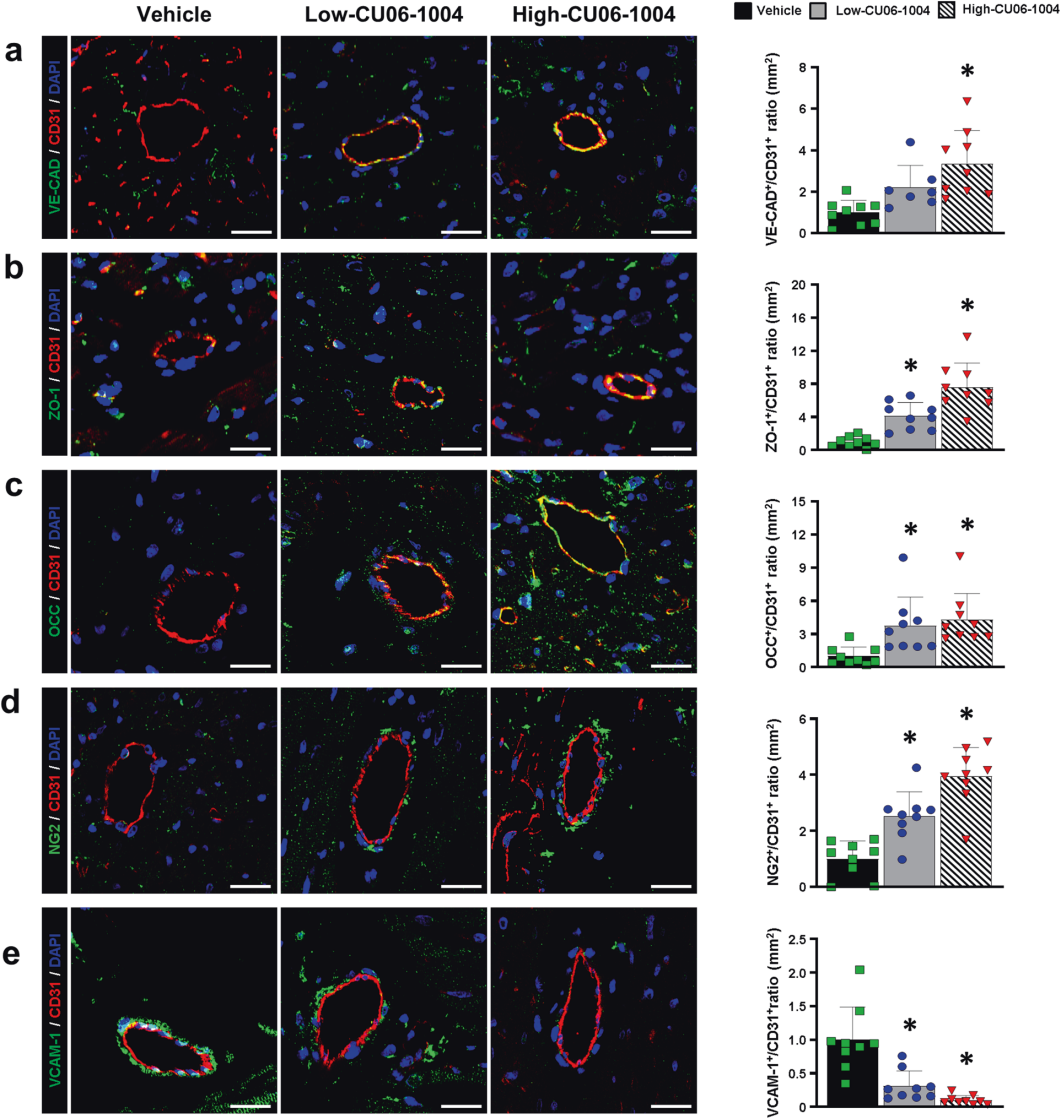
CU06-1004 protects the integrity of CMECs in I/R injury. **a**–**c** Representative images of CMEC junctions, stained for junction proteins such as (**a**) VE-CAD, (**b**) ZO-1, and (**c**) OCC, in the infarct zone and a summary of their quantification. VE-CAD, ZO-1, and OCC (green), CD31 (red), DAPI (blue). *n* = 7–9. *P < 0.05 vs. vehicle. Scale bars: 100 µm. **d** Representative images of pericytes enveloping CMECs in the infarct zone and a summary of their quantification. NG2 (green), CD31 (red), and DAPI (blue). *n* = 9. *P < 0.05 vs. vehicle. Scale bars: 100 µm. **e** Representative images of VCAM-1 related to inflammation in the infarct zone and a summary of their quantification. VCAM-1 (green), CD31 (red), and DAPI (blue). *n* = 9. *P < 0.05 vs. vehicle. Scale bars: 100 µm. Data are shown as the mean ± SEM.

Since CU06-1004 reduced cardiac edema by preventing vascular leakage into interstitial tissue, we investigated the stabilizing effect of CU06-1004 on the endothelial barrier by histologically evaluating the hearts 48 h after I/R injury. In the vehicle group, the expression of endothelial junction proteins was significantly reduced, with discontinuous staining of endothelial markers such as vascular endothelial cadherin (VE-CAD), zonula occludens-1 (ZO-1), and occludin (OCC), indicating an impaired endothelial barrier. However, both CU06-1004-treated groups had significantly higher expression of endothelial junction proteins in CD31-positive cells than the vehicle group (Fig. 2a–c). The extent and fraction of neural/glial antigen 2 (NG2)-positive pericytes, which normally cover the vessels, were undetectable in the vehicle group. However, the CU06-1004 groups exhibited a marked increase in pericyte coverage compared with the vehicle group (Fig. 2d). Pericytes are well known to envelop the outer surfaces of vessels to strengthen the endothelial junctions^18^. These findings suggest that CU06-1004 treatments maintain the stability of EC junctions following myocardial I/R injury.

**Fig. 3:**
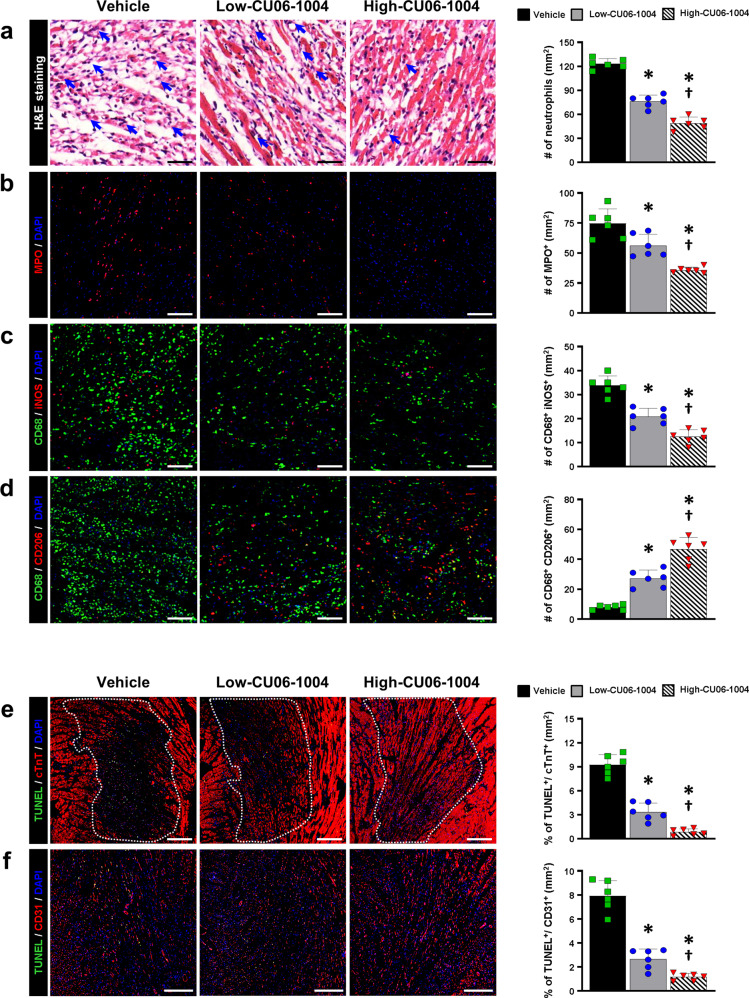
CU06-1004 prevents infiltration of inflammatory cells and apoptosis of CMs and CMECs. **a** Representative images of neutrophils in the infarct zone at 48 h after reperfusion and a summary of their quantification. The number of neutrophils was quantified by counting multilobed nuclei. *n* = 6. *P < 0.05 vs. vehicle. ^†^*P* < 0.05 vs. low CU06-1004. Scale bars: 100 µm. **b** Representative images of neutrophils stained with MPO in the infarct zone and a summary of their quantification. MPO (red) and DAPI (blue). *n* = 6. *P < 0.05 vs. vehicle. ^†^*P* < 0.05 vs. low CU06-1004. Scale bars: 100 µm. **c** Representative images of M1 macrophages in the infarct zone and a summary of their quantification. CD68 (green), iNOS (red) and DAPI (blue). *n* = 6. *P < 0.05 vs. vehicle. ^†^*P* < 0.05 vs. low CU06-1004. Scale bars: 100 µm. **d** Representative images of M2 macrophages in the infarct zone and a summary of their quantification. CD68 (green), CD206 (red), and DAPI (blue). *n* = 6. *P < 0.05 vs. vehicle. ^†^*P* < 0.05 vs. low CU06-1004. Scale bars: 100 µm. **e** Representative images of apoptotic CMs in the infarct zone and a summary of their quantification. TUNEL (green), cTnT (red) and DAPI (blue). *n* = 6. *P < 0.05 vs. vehicle. ^†^*P* < 0.05 vs. low CU06-1004. Scale bars: 200 µm. **f** Representative images of apoptotic CMECs in the infarct zone and a summary of their quantification. TUNEL (green), CD31 (red) and DAPI (blue). *n* = 6. *P < 0.05 vs. vehicle. ^†^*P* < 0.05 vs. low CU06-1004. Scale bars: 200 µm. Data are shown as the mean ± SEM.

Accumulating evidence suggests that myocardial I/R injury upregulates the expression levels of adhesion molecules on CMECs and the infiltration of inflammatory cells in the infarcted myocardial tissue, which releases cytokines, chemokines, and other proinflammatory stimuli^19–22^. In this study, we also demonstrated that the expression of the adhesion molecule VCAM-1 on the vessels was significantly decreased in the CU06-1004 groups compared with the vehicle group (Fig. 2e). To assess the extent of inflammatory cell infiltration in the infarcted tissue, we compared the numbers of neutrophils between the groups by counting multilobed nuclear and myeloperoxidase-positive cells. The CU06-1004 groups showed significant, dose-dependent reductions in neutrophil infiltration compared to the vehicle group (Fig. 3a, b). Furthermore, CU06-1004 treatment significantly reduced the infiltration of CD68 and iNOS double-positive M1 macrophages and increased the infiltration of CD68 and CD206 double-positive M2 macrophages compared with the vehicle in a dose-dependent manner (Fig. 3c, d). Finally, we quantified TUNEL-positive CMECs and CMs to evaluate the extent of apoptosis in cardiac cells. The low-CU06-1004 group had significantly fewer TUNEL-positive CMECs and CMs than the vehicle group, and the high-CU06-1004 group had even fewer (Fig. 3e, f and Supplementary Fig. 1a, b). Taken together, the evidence suggests that CU06-1004 can play a crucial role in cardiac function, potentially by stabilizing EC junctions and suppressing neutrophil-EC adhesion interactions, which are among the earliest signs of tissue dysfunction induced by I/R injury.

### CU06-1004 protects CMECs against H/R-induced injury

To investigate the mechanism through which CU06-1004 blocks vascular leakage, we cultured human CMECs in vitro with or without CU06-1004 under hypoxia/reperfusion (H/R) to mimic I/R injury (Supplementary Fig. 2a). Exposing CMECs to H/R-induced injury results in diverse microvascular alterations, including EC barrier disruption^23^, excessive ROS generation^24^, and an activated inflammatory response (including enhanced expression of adhesion molecules and inflammatory cytokines^25,26^. The MTT assay demonstrated that pretreatment with two concentrations of CU06-1004 (5 and 10 µg/mL) protected against H/R-induced cell death in human CMECs (Supplementary Fig. 2b) but not human CMs (Supplementary Fig. 2c). Interestingly, CM survival was improved when human CMECs and human CMs were cocultured with CU06-1004 under H/R conditions. However, this effect was negated in the absence of CU06-1004, indicating that human CMECs protected by CU06-1004 from H/R injury released cardioprotective factors that enhance human CM survival (Supplementary Fig. 2d). Furthermore, we performed ELISA to measure BNP and TNF-α in cell medium in which human CMECs and CMs had been cocultured. We observed that the levels of BNP, an indicator of CM injury, and TNF-α, a marker of inflammation, were increased after H/R injury. However, the levels of BNP and TNF-α in the CU06-1004-treated group were markedly reduced (Supplementary Fig. 2e, f). These findings suggest that CU06-1004 acts specifically on CMECs and that strategies enhancing CMEC survival and function can improve CM survival by mutual crosstalk between CMECs and CMs in myocardial I/R injury.

Since ROS are critical to the injury produced by in vitro H/R in cultured CMECs, we measured cellular ROS levels in CMECs and demonstrated that pretreatment with CU06-1004 suppressed H/R-induced ROS generation (Supplementary Fig. 2g). To verify the role of CU06-1004 in vascular integrity under H/R in vitro, we measured the permeability of the CMEC monolayer to FITC-dextran. CU06-1004 significantly blocked H/R-induced FITC-dextran leakage (Supplementary Fig. 2h). CU06-1004 significantly reduced the expression of adhesion molecules such as ICAM-1, VCAM-1, and E-selectin as well as proinflammatory cytokines such as MCP-1, TNF-α, and IL-1β (Supplementary Fig. 2i–n). These findings suggest that CU06-1004 reduces the production of ROS and the expression of inflammatory adhesion molecules and cytokines on CMECs in myocardial I/R injury.

### CU06-1004 improves long-term cardiac function after myocardial I/R injury

**Fig. 4:**
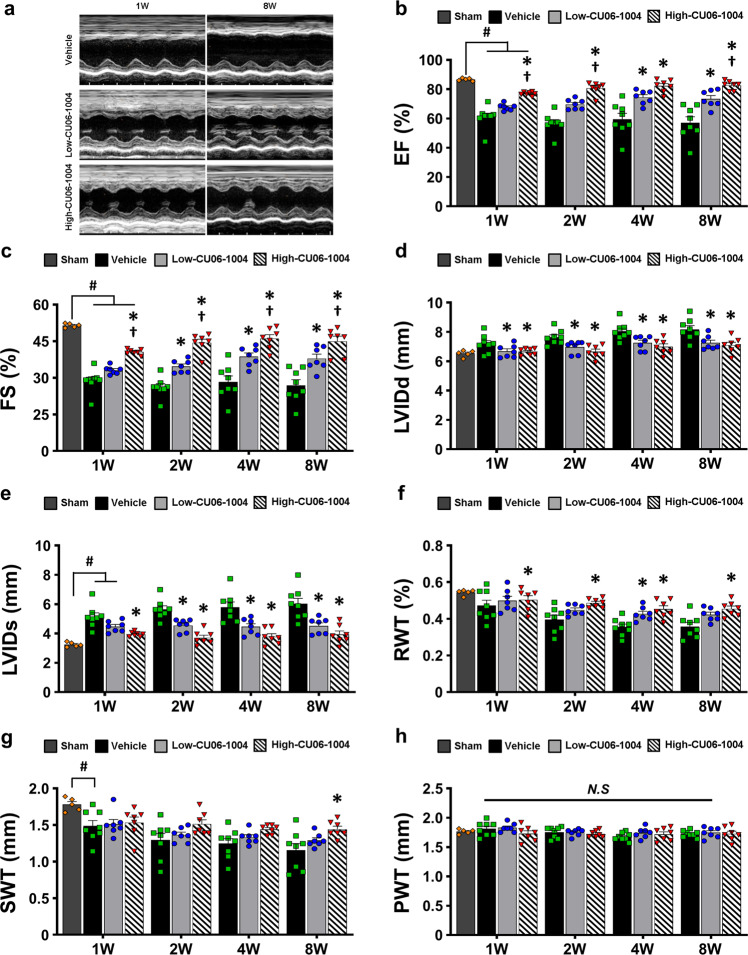
Protection of vascular integrity by CU06-1004 promotes long-term cardiac function after I/R injury. **a** Representative M-mode images of the three groups at 1 and 8 weeks after I/R. **b** Left ventricular ejection fraction (EF). **c** Left fractional shortening (FS). **d** Left ventricular internal diastolic dimension (LVIDd). **e** Left ventricular internal systolic dimension (LVISd). **f** Relative wall thickness (RWT). **g** Septal wall thickness (SWT). **h** Posterior wall thickness (PWT). *n* = 5–8. *P < 0.05 vs. vehicle. ^†^*P* < 0.05 vs. low-CU06-1004. ^#^*P* < 0.05 vs. sham. N.S. not significant. Data are shown as the mean ± SEM.

We performed serial echocardiography until 8 weeks after infarction to determine whether the early cardioprotective effect of CU06-1004 is sustained in the long term. The CU06-1004 treatments significantly improved the ejection fraction (EF) and fractional shortening (FS) compared with those of the vehicle group (Fig. 4a–c). Notably, in this 8-week period, the single high-dose treatment improved cardiac function more effectively than the repetitive low-dose treatments. Cardiac remodeling indices, such as left ventricular internal diameter at end diastole (LVIDd), left ventricular internal diameter at end-systole (LVIDs), and relative wall thickness (RWT), were also significantly decreased in the CU06-1004 groups compared with the vehicle group (Fig. 4d–f). Although posterior wall thickness (PWT) did not differ in all groups, septum wall thickness (SWT) was higher in the CU06-1004 groups than in the vehicle group (Fig. 4g, h).

**Fig. 5:**
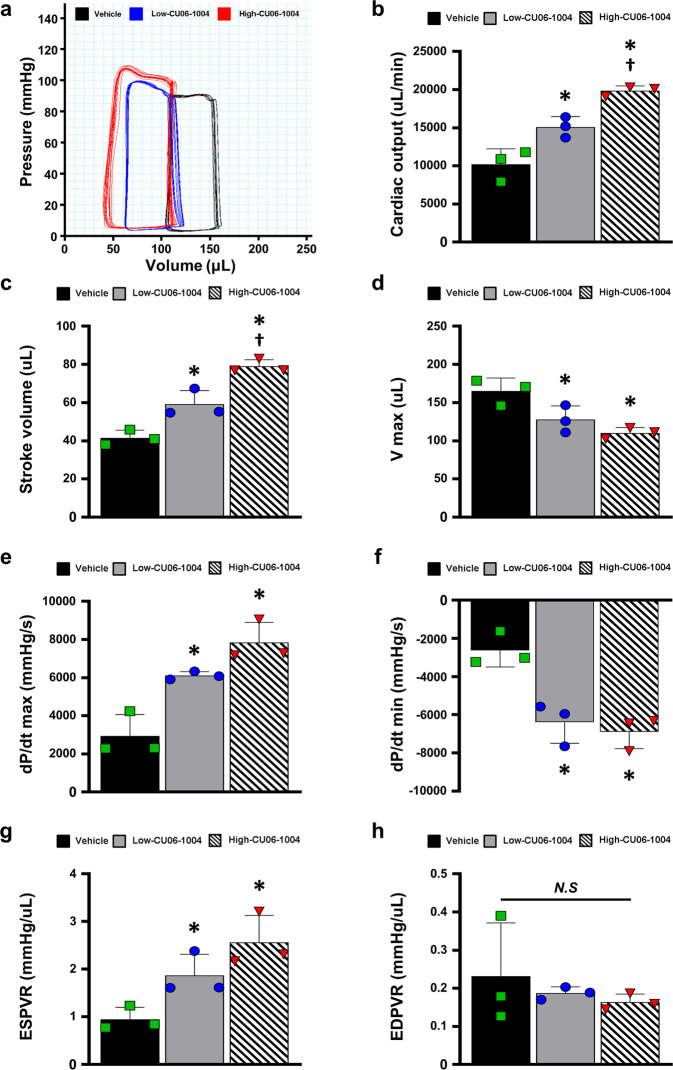
CU06-1004 improves hemodynamic cardiac contractibility. **a** Representative images of the hemodynamic pressure and volume (PV) curve under steady-state conditions at 8 weeks after I/R injury. **b** Cardiac output (CO). **c** Stroke volume (SV). **d** Maximum volume (V max) at end diastole. **e** Maximal rate of pressure changes during systole (dP/dt_max_). **f** Minimal rate of pressure changes during diastole (dP/dt_min_). **g** Slope of end-systolic pressure–volume relationship (ESPVR), indicating intrinsic cardiac contractility, as measured during transient inferior vena cava (IVC) occlusion. **h** Slope of end-diastolic pressure–volume relationship (EDPVR). *n* = 3. *P < 0.05 vs. vehicle. ^†^*P* < 0.05 vs. low-CU06-1004. N.S. not significant. Data are shown as the mean ± SEM.

Because cardiac function as evaluated by echocardiography is load-dependent, we further performed pressure–volume (PV) loop studies to determine intrinsic cardiac contractility at 8 weeks after MI. The parameters related to cardiac function, such as cardiac output (CO) and stroke volume (SV), showed that the CU06-1004 groups had a dose-dependent improvement in cardiac function compared with the vehicle group (Fig. 5a–c). Moreover, the maximum volume (V max), a cardiac remodeling index, was lower in the high-CU06-1004 group than in the vehicle group, indicating that CU06-1004 decreased adverse cardiac remodeling after I/R injury (Fig. 5d). However, the maximum pressure (P max) was not significantly different among the groups (Supplementary Fig. 3a). The maximum rate of pressure change (dP/dt_max_) and minimum rate of pressure change (dP/dt_min_) were significantly increased in the CU06-1004 groups compared with the vehicle group (Fig. 5e, f). These results suggest that the administration of CU06-1004 improves cardiac function under steady-state conditions. Load-independent cardiac contractility was assessed via temporary occlusion of the inferior vena cava, and this assessment showed that the CU06-1004 groups had significantly higher contractile function than the vehicle group, including a steeper end-systolic pressure–volume relationship (ESPVR) (Fig. 5g and Supplementary Fig. 3b–d). The slope of the end-diastolic pressure–volume relationship (EDPVR) was comparable in all groups (Fig. 5h and Supplementary Fig. 3b–d). As confirmed by echocardiography, these results suggest that treatment with CU06-1004, especially in a single high-dose treatment, improves cardiac function and reduces adverse remodeling in I/R-injured hearts.

### CU06-1004 ameliorates the adverse microenvironment of infarcted hearts after I/R injury

**Fig. 6:**
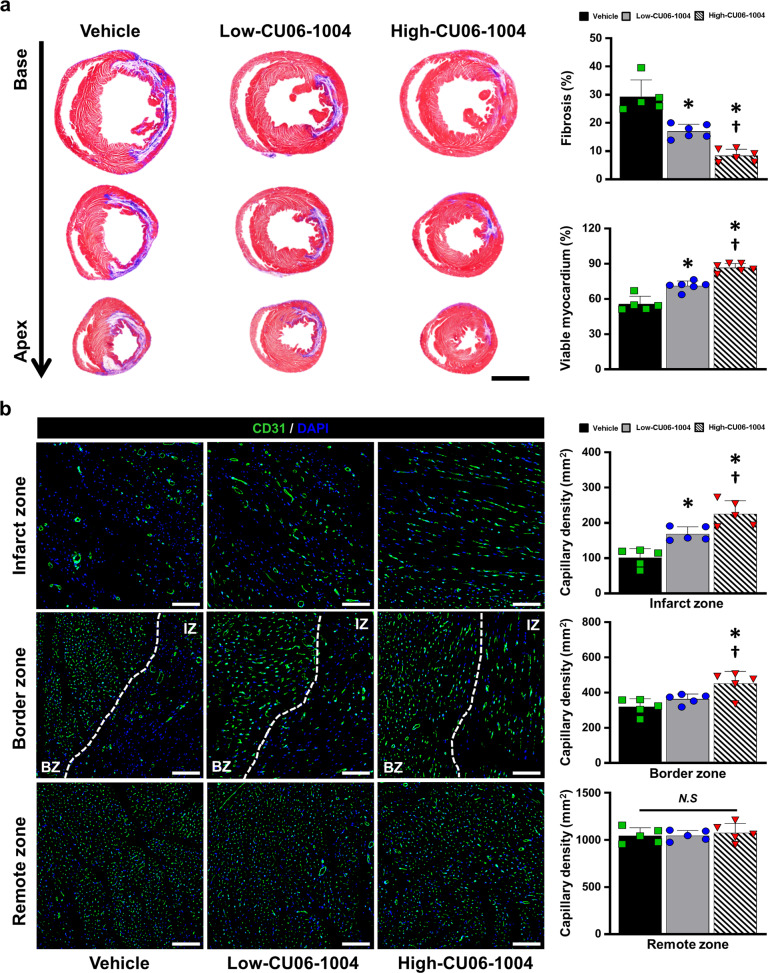
CU06-1004 reduces cardiac fibrosis and increases capillary density. **a** Representative images of Masson’s trichrome staining at 8 weeks and quantitative summary showing the percentage of fibrosis and viable myocardium. *n* = 5–6. *P < 0.05 vs. vehicle. ^†^*P* < 0.05 vs. low CU06-1004. Scale bars: 2000 µm. **b** Representative images of capillaries stained for CD31 (green) in the infarct zone, border zone, and remote zone at 8 weeks and a summary of their quantification. *n* = 5. *P < 0.05 vs. vehicle. ^†^*P* < 0.05 vs. low CU06-1004. Scale bars: 100 µm. N.S. not significant. Data are shown as the mean ± SEM.

In infarcted hearts, fibroblast infiltration triggers fibrosis by secreting collagen and other matrix proteins, which impair cardiac function. To verify the therapeutic effects of CU06-1004 on fibrosis, we performed Masson’s trichrome staining of post-MI hearts at 8 weeks and showed that CU06-1004 reduced the percentage of fibrosis and increased the amount of viable myocardium in the LV wall in a dose-dependent manner compared with the vehicle (Fig. 6a and Supplementary Fig. 4a). Furthermore, we performed immunohistochemical staining for CD31 to assess capillary density; this assay also showed a dose-dependent increase in capillary density in both the border and infarct zones of the injured hearts of the CU06-1004 groups with the vehicle group (Fig. 6b). These findings suggest that CU06-1004 treatment ameliorates the harsh microenvironment in infarcted hearts after I/R injury, which might be associated with the strengthening of CMEC junctions and suppression of edema, inflammation, and apoptosis of parenchymal cells in the early stage of I/R injury.

## Discussion

Although many potential cardioprotective therapies against myocardial I/R injury have been investigated for their effects on cellular and intracellular targets, no satisfactory outcomes have been reported in clinical trials for AMI patients^27–31^. These results might be attributable to the emphasis on the role of CMs, even though CMECs are also critical mediators of myocardial dysfunction after I/R injury^10,25^. To the best of our knowledge, this study is the first to identify that CU06-1004 can improve cardiac function by inhibiting CMEC hyperpermeability and subsequently reducing capillary plugging and infiltration by neutrophils in infarcted hearts.

Tissue damage in myocardial I/R injury is triggered by the overproduction of ROS, which, in turn, leads to CMEC dysfunction characterized by reduced NO bioavailability, elevated expression of proinflammatory factors, and disrupted endothelial structure^25^. Excess vascular hyperpermeability and tissue edema impair the ability of the heart to pump efficiently. In addition, the overexpression of adhesion molecules such as VCAM-1, ICAM-1, and E-selectin on the surfaces of activated CMECs boosts the recruitment of neutrophils to ischemic tissue and worsens I/R injury^26,32–34^. Therefore, the strategy of blocking the pathological process of I/R injury at its first step by enhancing the integrity of CMECs is a very attractive and promising approach for preventing I/R injury.

Herein, CU06-1004, whose efficacy and safety have been established previously in preclinical disease models related to endothelial dysfunction, was established as a vascular leakage blocker; its principal mode of action appears to be mediated by anti-inflammatory effects based on suppressing NF-κB activation and by anti-permeability effects occurring through activation of the small GTPase Rac and translocation of cortactin in ECs^11,12^. In the infarcted heart, CU06-1004 protected intrinsic junctions and structures of CMECs and maintained pericyte coverage surrounding the CMECs, which plays a critical role in the stabilization, function, and proliferation of CMECs through mutual interaction^18^. As a natural consequence, CU06-1004 decreased not only inflammatory responses associated with the infiltration of neutrophils and M1 macrophages but also apoptosis of CMs and CMECs in infarcted hearts 48 h after I/R injury. Interestingly, these initial favorable effects ameliorated the harsh microenvironment of infarcted hearts with reduced fibrosis and increased capillary density, resulting in improved cardiac function.

CMECs not only serve as a protective barrier between the lumen and the smooth muscle of vessels but also regulate the function of CMs^35,36^. Several reports have demonstrated that CMECs provide cytoprotective signals for CMs under H/R conditions^37–39^. ROS activate NRG-1β/erbB4 paracrine signaling in CMECs, which promotes the survival of CMs as a cardiac adaptation to oxidative stress^40^. NO released from CMECs diffuses into CMs and affects the contractility of muscle fibers via effects on Ca^2+^ storage in the sarcoplasmic reticulum (SR)^41^. In this study, our results showed that CU06-1004 had a direct effect on CMECs but not on CMs. Nevertheless, CU06-1004 treatment improved CM survival after H/R-induced injury when CMECs were cocultured with CMECs for 24 h using a Transwell assay. These findings suggest that strategies enhancing CMEC survival and function after myocardial I/R injury can improve CM survival by mutual crosstalk between CMECs and CMs, providing novel insight into the management of AMI patients treated with primary PCI.

We also determined the therapeutic window of CU06-1004 for treating myocardial I/R injury by comparing the effects of two different CU06-1004 regimens on vascular integrity at 48 h after reperfusion; these regimens, varying in dose and frequency, comprised repetitive low-dose treatment (1 mg/kg, twice at 24 h intervals) and a single high-dose treatment (5 mg/kg, once before reperfusion). Although both strategies ensured the stability of CMEC junctions and pericyte coverage in the infarcted hearts 2 days after I/R injury, single high-dose treatment with CU06-1004 was preferable to repetitive low-dose treatment and significantly improved long-term cardiac function without depending on a loading dose. These findings suggest that therapeutic measures against reperfusion injury should be started as early as possible after reperfusion because most cell death occurs during the first few minutes of reperfusion.

**Fig. 7:**
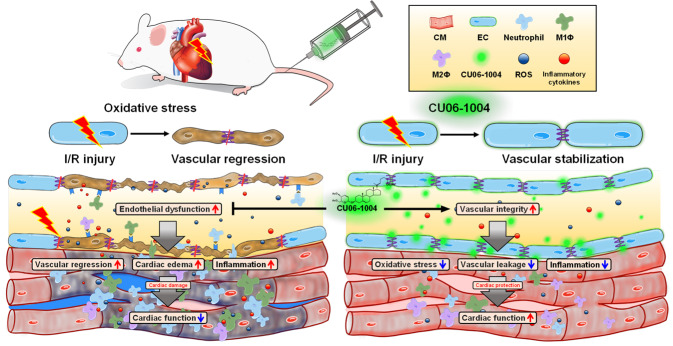
Schematic diagram of the underlying therapeutic mechanisms of CU06-1004. CU06-1004 enhances vascular integrity and improves cardiac function by preventing lethal myocardial I/R injury. CM cardiomyocyte, EC endothelial cell, ROS reactive oxygen specie.

In conclusion, novel treatment approaches are required as potential adjuncts to current reperfusion strategies for AMI to provide further reductions in morbidity and mortality. The coronary microcirculatory function is an important determinant of clinical outcomes, and damage to CMECs by oxidative stress plays a crucial role in the pathological process of the coronary no-reflow phenomenon, such as capillary blockage by neutrophils and platelets due to microvascular leakage and mechanical compression associated with myocardial edema. This study demonstrates that early intervention with CU06-1004 enhances vascular integrity and improves cardiac function by suppressing edema and inflammation in myocardial I/R injury (Fig. 7). From a clinical perspective, the pharmacological action of CU06-1004 can be applied to prevent the no-reflow phenomenon, which is a major therapeutic target to improve the clinical outcomes of AMI patients^42^.

## Supplementary information

Supplementary information
