# Supplementary material for: CU06-1004 enhances vascular integrity and improves cardiac remodeling by suppressing edema and inflammation in myocardial ischemia–reperfusion injury
Source: Exp Mol Med. 2022 Jan 7;54(1):23–34. doi: 10.1038/s12276-021-00720-w (PMC8814060; doi:10.1038/s12276-021-00720-w)

Supplementary Information

CU06-1004 enhances vascular integrity and improves cardiac remodeling by suppressing edema and inflammation in myocardial ischemia-reperfusion injury

Running title: Therapeutic potential of CU06-1004 in I/R injury

Haiying Zhang[1,2,*], Hyeok Kim[3,*], Bong Woo Park[3], Minyoung Noh[1], Yeomyeong Kim[1], Jeongeun Park[1], Jae-Hyun Park[3], Jin-Ju Kim[3], Woo-Sup Sim[3], Kiwon Ban[5], Hun-Jun Park[3,4,#], Young-Guen Kwon[1,#]

[1]Department of Biochemistry, College of Life Science and Biotechnology, Yonsei University, Seoul 120-749, Republic of Korea

[2]R&D Department, Curacle Co. Ltd, Seongnam-si, Republic of Korea

[3]Department of Medical Life Science, College of Medicine, The Catholic University of Korea, Seoul 06591, Republic of Korea

[4]Division of Cardiology, Department of Internal Medicine, The Catholic University of Korea, Banpo-daero 222, Seocho-gu, Seoul, 137701, Republic of Korea

[5]Department of Biomedical Sciences, City University of Hong Kong, Kowloon Tong 999077, Hong Kong.

* These authors contributed equally.

[#] Address for correspondence

Hun-Jun Park: M.D, Ph.D, Division of Cardiology, Seoul St. Mary's Hospital, The Catholic University of Korea, Seoul 137-701, Republic of Korea. Email: cardioman@catholic.ac.kr; Young-Guen Kwon: Ph.D, Department of Biochemistry, College of Life Science and Biotechnology, Yonsei University, Seoul 120-749, Republic of Korea. Email: ygkwon@yonsei.ac.kr

Supplementary figures

Supplementary Fig. 1. Integrity of vessels protected by CU06-1004 results in anti-apoptosis of CMs and CMECs.

a High magnification images of Figure 3E. TUNEL (green), cTnT (red), DAPI (blue). Scale bars: 100 µm. **b** High magnification images of Figure 3F. TUNEL (green), CD31 (red), DAPI (blue). Scale bars: 100 µm.

Supplementary Fig. 2. CU06-1004 prevents cell death, overproduction of ROS, hyperpermeability, and inflammation of CMECs in H/R injury.

a Schematic illustration of the experimental hypoxia and reoxygenation setup to stimulate I/R in vitro. Human CMECs were exposed to vehicle and CU06-1004 and cultivated for 16h under hypoxia. The cells were then reoxygenated for 24h. **b** Viability of human CMECs was assessed by an MTT. n=6. *$p<0.05$ vs. Normoxia. †$p<0.05$ vs. Vehicle. **c** Viability of human CMs was assessed by an MTT. n=6. *$p<0.05$ vs. Normoxia. †$p<0.05$ vs. Vehicle. **d** Human CMs was co-cultured with the human CMECs then subject to H/R and cell viability was determined by MTT. n=4. *$p<0.05$ vs. Normoxia. †$p<0.05$ vs. Vehicle. **e-f** ELISA assay for **(e)** BNP and **(f)** TNF-α level in co-cultured medium of human CMECs and CMs. n=5. *$p<0.05$ vs. Normoxia. †$p<0.05$ vs. Vehicle. **g** Generation of ROS was measured by DCF-DA staining. n=6-8. *$p<0.05$ vs. Normoxia. †$p<0.05$ vs. Vehicle. **h** The integrity of human CMEC was assessed by FITC-Dextran permeability assay. n=6. *$p<0.05$ vs. Normoxia. †$p<0.05$ vs. Vehicle. **i-k** qRT-PCR analysis of relative mRNA expression associated with inflammatory adhesion molecules of human CMEC, such as **(i)** ICAM-1, **(j)** VCAM-1, **(k)** E-selectin. **l-n** qRT-PCR analysis of relative mRNA expression associated with inflammatory factors of human CMEC, such as **(l)** MCP-1, **(m)** TNF-α, **(n)** IL-1β. The y axis represents relative mRNA expression of target genes to glyceraldehyde-3-phosphate dehydrogenase (GAPDH). n=4-8. *$p<0.05$ vs. Normoxia. †$p<0.05$ vs. Vehicle. Data are shown as the mean ± S.E.M.

Supplementary Fig. 3. Hemodynamic instinct cardiac contractibility.

a Maximal pressure at end-systole (P max). n=3. N.S: not significant. **b-d** Representative image of Intrinsic cardiac function in **(b)** Vehicle group, **(c)** Low-CU06-1004 group, **(d)** High-CU06-1004 group.

Supplementary Fig. 4. Cardiac fibrosis attenuated by CU06-1004.

a Masson's trichrome images of all animals in each group. n=5.

Supplementary information accompanies the manuscript on the Experimental & Molecular Medicine` website (http://www.nature.com/emm/)

Supplementary Fig. 1

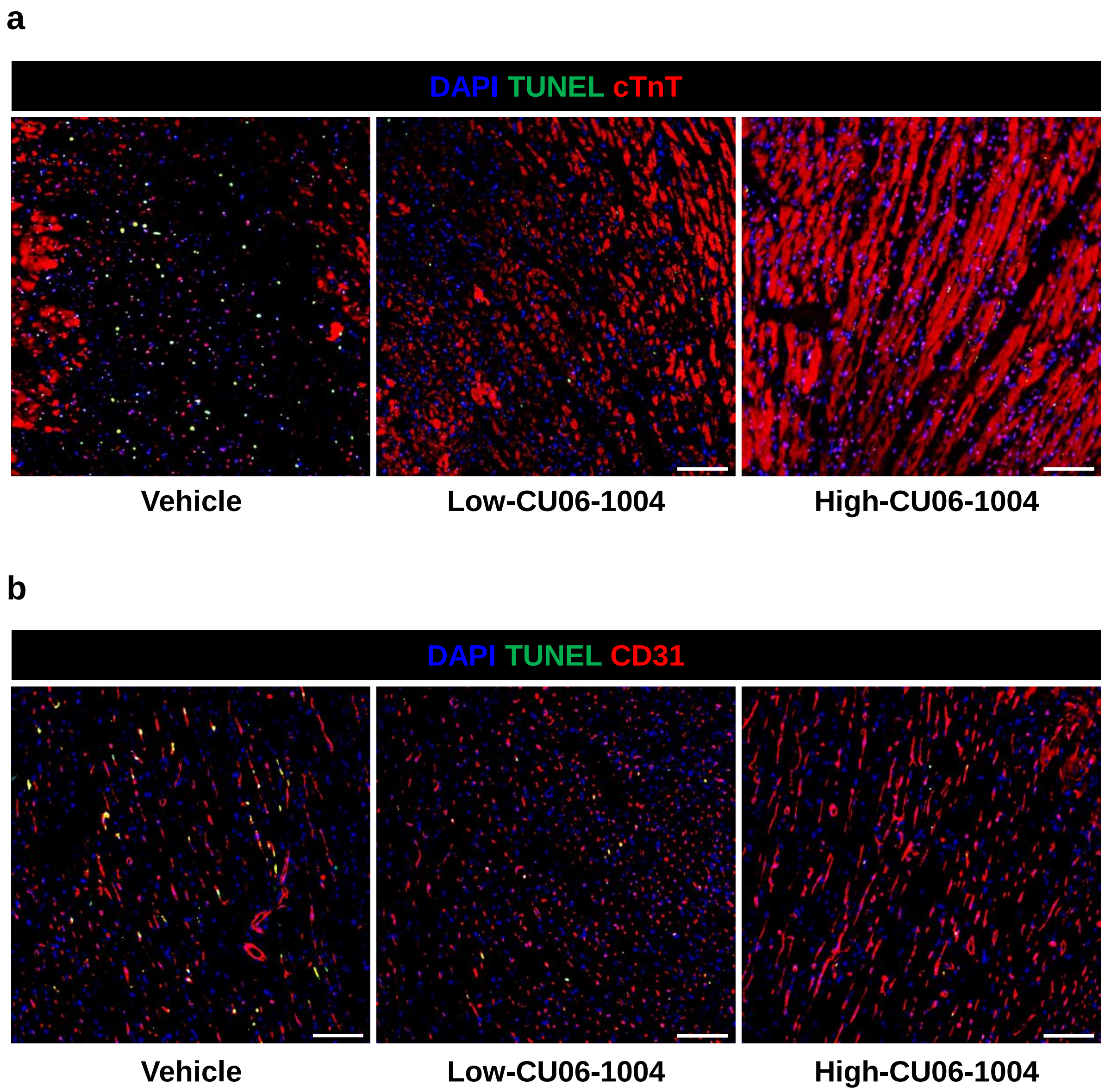

Supplementary Fig. 2

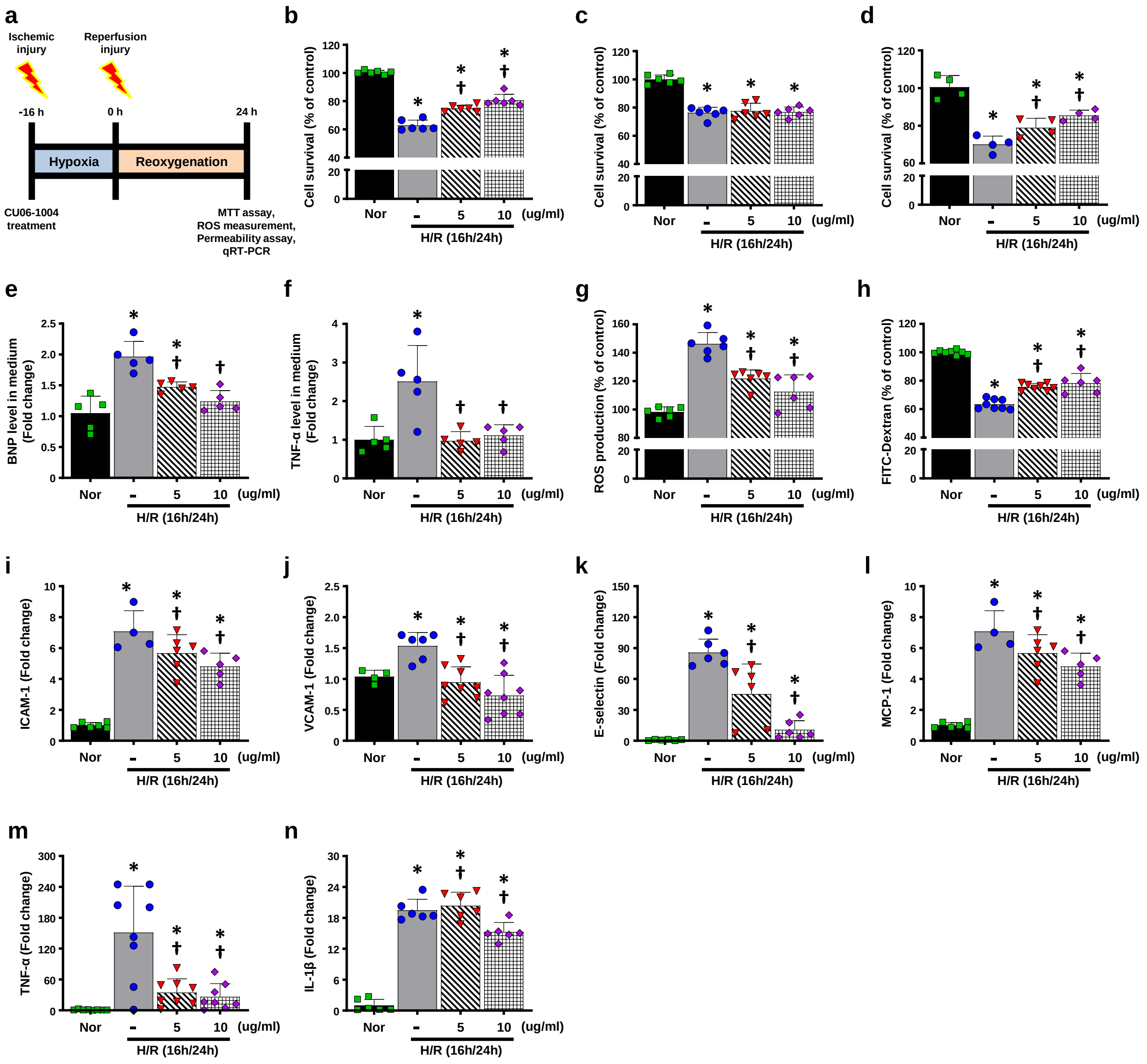

Supplementary Fig. 3

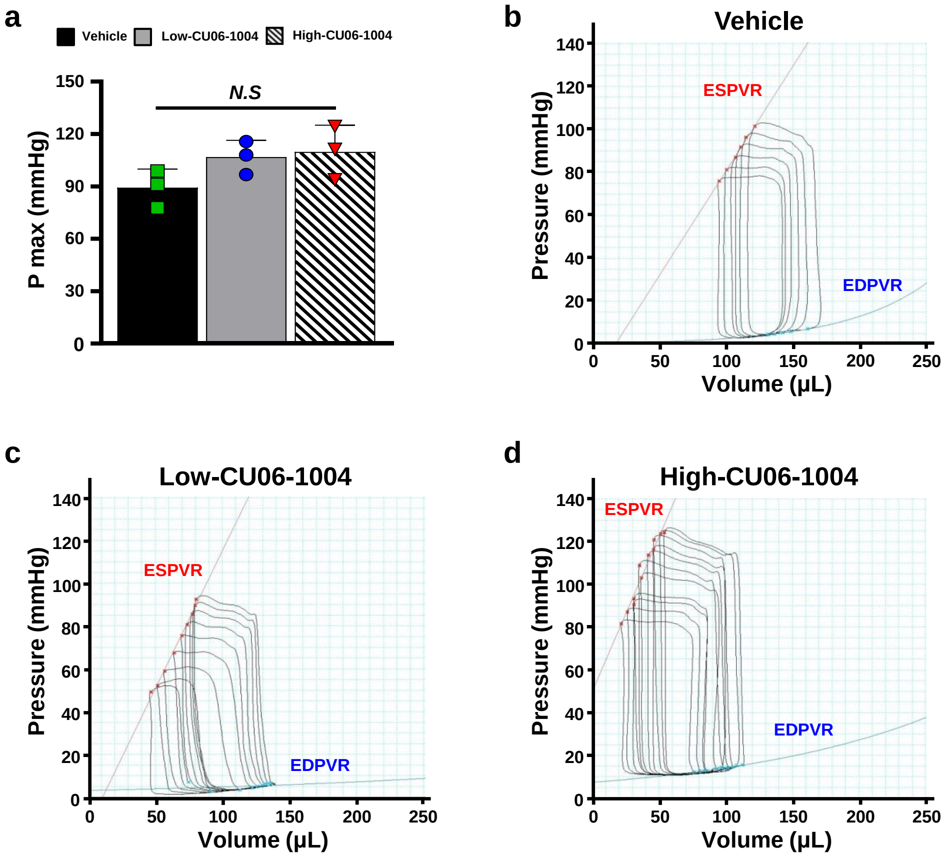

Supplementary Fig. 4

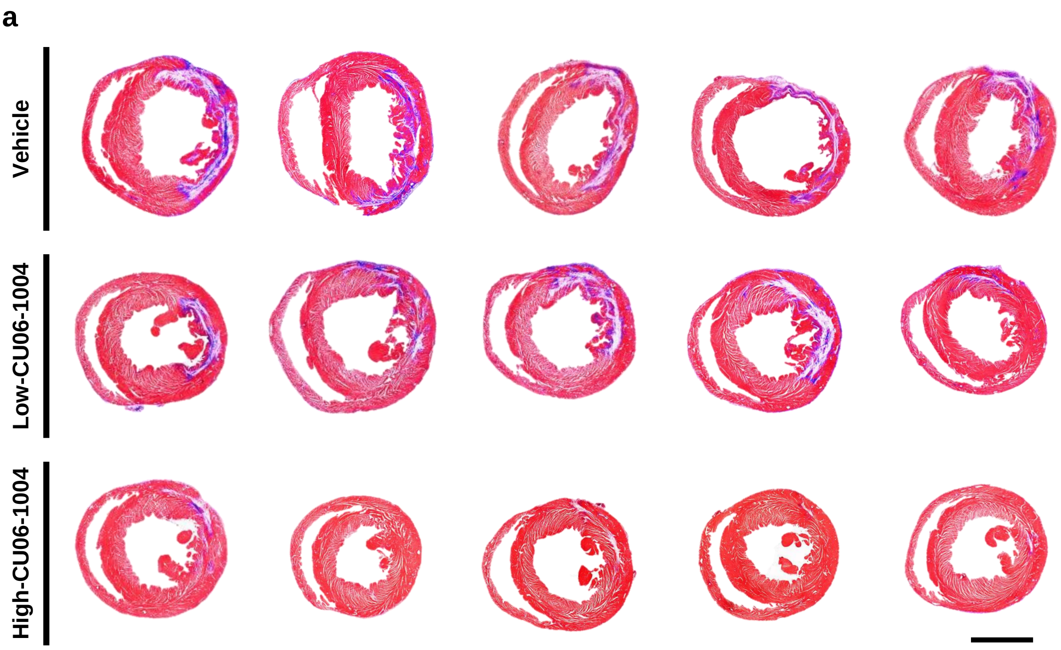

Supplement: Supplementary file 1 — Supplementary information [file 12276_2021_720_MOESM1_ESM.pdf]
